# Identification of amplified and highly expressed genes in amplicons of the T-cell line huT78 detected by cDNA microarray CGH

**DOI:** 10.1186/1476-4598-4-5

**Published:** 2005-01-18

**Authors:** Bárbara Meléndez, Beatriz Martínez-Delgado, Marta Cuadros, Victoria Fernández, Ramón Díaz-Uriarte, Javier Benítez

**Affiliations:** 1 Human Genetics Department, Spanish National Cancer Centre (CNIO), c/Melchor Fernéndez Almagro 6, 28029-Madrid, Spain; 2 Bioinformatics Unit, Spanish National Cancer Centre (CNIO), c/Melchor Fernéndez Almagro 6, 28029-Madrid, Spain

## Abstract

**Background:**

Conventional Comparative Genomic Hybridization (CGH) has been widely used for detecting copy number alterations in cancer and for identifying regions containing candidate tumor responsible genes. Recently, several studies have shown the utility of cDNA microarray CGH for studing gene copy changes in various types of tumors. However, no such studies on T-cell lymphomas have been performed. To date T-cell lymphomas analyzed by the use of chromosome CGH have revealed only slight copy number alterations and not gene amplifications.

**Results:**

In the present study, we describe the characterization of three amplicons of the T-cell line huT78 located at 2q34-q37, 8q23-q24 and 20p, where new amplified and overexpressed genes are found. The use of a cDNA microarray containing 7.657 transcripts allowed the identification of certain genes, such as *BCLX*, *PCNA*, *FKBP1A*, *IGFBP2 *and *cMYC*, that are amplified, highly expressed, and also contained in the amplicons on 20p and 2q. The expresion of these genes was analyzed in 39 T-cell lymphomas and 3 other T-cell lines.

**Conclusion:**

By the use of conventional CGH and CGH and expression cDNA microarrays we defined three amplicons in the T-cell line huT78 and identified several novel gene amplifications (*BCLX*, *PCNA, FKBP1A, IGFBP2 *and *cMYC*). We showed that overexpression of the amplified genes could be attributable to gene dosage. We speculate that deregulation of those genes could be important in the development of T-cell lymphomas and/or in the maintenance of T-cell lines.

## Background

Gene amplification plays an important role in the progression and initiation of many solid tumors, as is the case of breast cancer where amplification of the genes *ERBB2 *(17q12), c*MYC *(8q24), and *CCND1 *(11q13) are found in 10–25% of breast tumors. Amplifications are revealed by Comparative Genomic Hybridization (CGH) in small chromosome areas (restricted to 2–10 Mb) where DNA copy number increases from more than 5 to 10-fold. Within these amplicons it is possible to identify critical amplified genes that are also overexpressed: this is the case for *cMYC *(8q24.12), *ERBB2 *(17q12-q21), *MDM2 *(12q14.3-q15) or *BCL2 *(18q21.3) [1-3]. Recently array-based CGH on cDNA microarrays has been used to investigate the genomic alterations with high resolution. Using this technique exhaustive analysis of the 17q12 and 17q23 amplicons in breast cancer has led to the identification of other genes that are also contained in the amplicons and whose overexpression could also be attributable to gene amplification [4-8]. Other recent studies in prostate cell lines [9] and in neuroblastoma tumors [10,11] have also shown the utility of cDNA microarray CGH in defining amplicon boundaries and in analyzing the complexity of the amplicons. Tumor-related genes contained in amplicons are identified in these works due to the posibility of correlating gene expression to gene dosage data. To date, however, no amplifications have been described in primary tumors or cell lines derived from T-cell lymphomas [12,13].

## Methods

The cutaneous T-cell line huT78 was obtained from American Type Culture Collection (Rockville, MD) and cells were grown under recommended culture conditions. High molecular weight DNA was extracted and CGH was performed as described previously [12,14].

In order to define regions of high-level amplification and to identify amplified and highly expressed genes contained in the amplicons, we performed cDNA microarray experiments on T-cell line huT78 to obtain expression and genomic profiles of the amplicons. We used the CNIO Oncochip (v1.1a) containing 7.657 different sequence-validated I.M.A.G.E cDNA clones (Research Genetics; Huntsville, AL) -some of them duplicated to reach a total of 11.718 spots- that represent known genes and expressed sequence tags (ESTs) related to the tumoral process, and tissue specific genes. A complete list can be found at .

Genomic DNA hybridizations on microarrays were performed using DNA extracted from the T-cell line huT78 and from the blood of a control donor used as a reference. Genomic DNAs were *Alu*I and *Rsa*I digested, labeled with Cy5 (huT78) and Cy3 (control) using BioPrime labeling kit (Life Technologies, Inc., Gaithersburg, MD), and hybridized on microarrays at 50°C for 14–16 h, as previously described [15]. Post-hybridization washes were performed and microarrays were scanned using a GenePix scanner (Axon Instruments, Foster City, CA). Fluorescence ratios Cy5/Cy3 were obtained and normalized by adjusting these ratios to a normalized factor so that the median of the ratios of all spots in the array equals 1. Only measurements with fluorescence intensities higher than two times the sum of the background averages' of both fluorochromes (Cy3 and Cy5) were considered reliable. Logarithms of the fluorescence ratios (log_2 _values) were calculated and used for the analysis. The CNIO Oncochip contains 7.657 different clones, of which 3.079 are replicated at least twice: thus, we removed and averaged the replicates by using an in-house developed preprocessing tool [16].

Expression data was also obtained from huT78 cells and from magnetically isolated T lymphocytes obtained from the pooled peripheral blood of 5 anonymous donors that were used as a control. T lymphocytes were isolated by using either magnetic microbeads conjugated to monoclonal mouse anti-human CD3 antibodies purchased from Miltenyi Biotec Inc. (Auburn, CA), or magnetic depletion of non-T-cells with a cocktail of antibodies using the Pan T-cell Isolation Kit (Miltenyi Biotec Inc.). Total RNAs were extracted with Tri Reagent (Molecular Research Center, Cincinnati, OH) following the manufacturer's instructions and amplified using a T7-based method, as previously described [17,18]. Briefly, 5 μgr of total RNA were used to produce double-stranded cDNA (Superscript Choice System, Life technologies Inc.) and amplification of mRNAs was performed using the Megascript T7 *in vitro *transcription kit (Ambion, Austin, TX) following manufacturer's recommendations. A pool of aRNAs obtained from the Universal Human RNA (Stratagene, La Jolla, CA) was used as a standard reference in all hybridizations. Test or reference amplified RNAs (aRNAs) were labeled with fluorescent Cy5 and Cy3, respectively, as reported [18] and hybridized on microarrays at 42°C for 15 hours. Fluorescence ratios (Cy5/Cy3) were normalized and filtered for genomic data. The Cy5/Cy3 ratios obtained in the cell line hybridization were then compared to those obtained in control T lymphocytes hybridization.

In order to analyze if the candidate genes contained in the amplicons are altered in primary tumors and other cell lines, we analyzed expression data obtained in a previous study [19] using tumor samples from 39 primary T-cell lymphomas and 3 cell lines (Jurkat, Molt 16 and Karpas 45). Sample description and clinical details are specified in the work from Martinez-Delgado et al. [19]. All the tumors were diagnosed according to the World Health Organization classification criteria, and all individuals had given official consent.

## Results

### CGH studies

**Figure 1:**
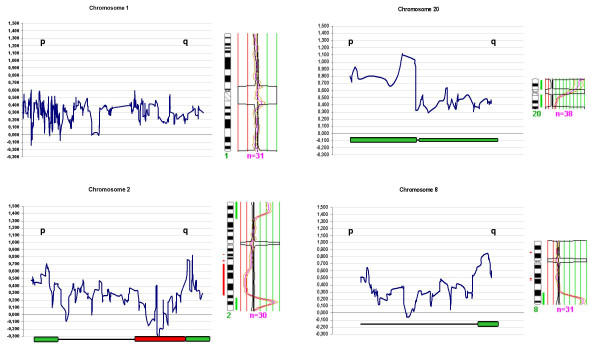
**CGH and genomic profiles of chromosomes 1, 2, 8 and 20 of the huT78 cell line**. Average of the log2 genomic values over 3 neighbouring genes are plotted in the figure as a function of the location of the clones according to EnsEMBL database. On the right of each graph, CGH profiles show the number of chromosomes analysed (n) and the average profile of the metaphases studied with a 99% interval of confidence. Red and green bars at both sides of each ideogram indicate gains or losses. Only 4 chromosomes are shown in the figure, chromosome 1 did not present DNA amplification, neither by CGH nor by microarray experiments, whilst chromosomes 2, 8 and 20 showed high-level DNA amplification at 2q34-q37, 8q23-q24 and 20p. On the bottom of each graph amplicons are represented (green bars) and the gain or loss regions (green and red bars, respectively). **p **and **q **arms are also indicated.

CGH carried out in huT78 cells revealed three high-level gains at 2q34-q37, 8q23-q24 and 20p, showing copy number gains higher than 5 fold (Figure 1). Low-level gains and losses of whole chromosomes/chromosomal regions were also detected in other chromosomes, with the exception of chromosomes 1, 21 and 22 that did not show copy number alterations.

### Expression and copy number profiling

**Figure 2:**
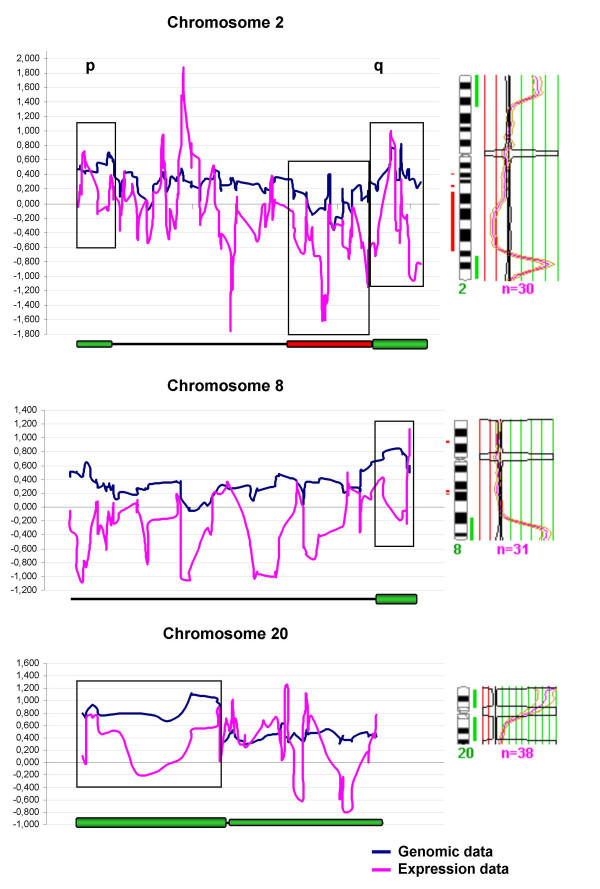
**Genomic and expression profiles of chromosomes 2, 8 and 20 of the huT78 cell line**. Chromosomes presenting regions of high level amplification are shown in the figure. Genomic and expression microarray data (averages of log_2 _values over 3 neighbouring genes) are plotted as a function of the location of the clones. At the foot of each graph amplicons are represented (gross green bars) along with the gain or loss regions (green and red bars, respectively). **p **and **q **arms are also indicated.

Expression and copy number profiling across each chromosome were performed using data from a total of 4.229 tumor-related genes or ESTs that had a map position and an identity confirmed by in-house sequencing. Map positions of the cDNA clones were obtained from the EnsEMBL database . According to this database, clones were ordered along the chromosomes and their expression and genomic values (See additional data file 1 for the raw data used to perform this analysis) were plotted as a function of their location to obtain chromosomal genomic and expression profiles (Figure 2).

Only thirty genes that showed high-level genomic gains were also highly expressed in the cutaneous T-cell line huT78 (Table 1). Genes were defined as significantly up-regulated (or down-regulated) if the difference in ratio to the control was at least two-fold (log_2 _[ratio expression data] ≥ +/-1). Cut-off levels for genomic data were defined at more than 1.7, a significantly high value to assure that the gene is gained at least 4-fold (log_2 _[genomic data] ≥ 0,8) (data from FISH studies). Within these thirty genes that were gained and overexpressed in the cell line, 5 of them were located in amplicons of chromosome 2 (*XRCC5*, *IGFBP2 *and *PSMB3*) and chromosome 20 (*FKBP1A *and *BCL2L1*) revealed by conventional CGH.

**Table 1:** Genes highly gained and overexpressed in huT78 cell line.

**Unigene ID**	**Gene Symbol**	**G**	**E**	**Log_2_(G)**	**Log_2_(E)**	**Cytogenetic Location**	**Gene**
Hs.76884	*ID3*	1,857	2,343	0,893	1,228	1p36.13-p36.12	inhibitor of DNA binding 3, dominant negative helix-loop-helix protein
**Hs.84981**	***XRCC5***	**2,04**	**4,044**	**1,029**	**2,016**	**2q35**	**X-ray repair complementing defective repair in Chinese hamster cells 5**
**Hs.162**	***IGFBP2***	**2,563**	**4,544**	**1,358**	**2,184**	**2q33-34**	**insulin-like growth factor binding protein 2**
**Hs.82793**	***PSMB3***	**1,794**	**3,874**	**0,843**	**1,954**	**2q35**	**proteasome subunit, beta type, 3**
Hs.174007	*VHL*	3,119	2,044	1,641	1,031	3p26-p25	von Hippel-Lindau syndrome
Hs.55173	*CELSR3*	1,862	2,647	0,897	1,404	3p24.1-p21.2	cadherin, EGF LAG seven-pass G-type receptor 3
Hs.180145	*HSPC030*	1,777	2,947	0,829	1,559	3	HSPC030 protein
Hs.350266	*ARGBP2*	1,823	2,028	0,866	1,020	4q35.1	Arg/Abl-interacting protein ArgBP2
Hs.179565	*MCM3*	1,791	4,378	0,841	2,130	6p12	MCM3 minichromosome maintenance deficient 3
Hs.278589	*GTF2I*	1,886	2,379	0,915	1,251	7q11.23	general transcription factor II, i
Hs.167246	*POR*	2,007	2,609	1,005	1,384	7q11.2	P450 (cytochrome) oxidoreductase
Hs.61762	*HIG2*	1,885	3,152	0,915	1,656	7q32.2	hypoxia-inducible protein 2
Hs.274424	*SAS*	1,743	4,944	0,802	2,306	9p24.1-p23	N-acetylneuraminic acid phosphate synthase; sialic acid synthase
Hs.184793	*DKFZP434F195*	1,78	2,125	0,832	1,087	9	DKFZP434F195 protein
Hs.18910	*POV1*	1,78	3,702	0,832	1,888	11p11.2-p11.1	prostate cancer overexpressed gene 1
Hs.91877	*THRSP*	6,491	3,095	2,698	1,630	11q13.5	thyroid hormone responsive
Hs.180628	*DNM1L*	1,758	2,715	0,814	1,441	12p12.3	dynamin 1-like
Hs.76294	*CD63*	1,747	6,165	0,805	2,624	12q12-q13	CD63 antigen (melanoma 1 antigen)
Hs.247888	-	2,284	3,201	1,192	1,679	16	
Hs.12303	*SUPT6H*	1,742	2,819	0,801	1,495	17q11.2	suppressor of Ty 6 homolog (S. cerevisiae)
Hs.296281	*ILF1*	1,754	3,146	0,811	1,653	17q25	interleukin enhancer binding factor 1
Hs.75716	*SERPINB2*	2,358	3,767	1,238	1,913	18q21.3	serine (or cysteine) proteinase inhibitor, clade B (ovalbumin), member 2
Hs.8372	*UQCR*	2,046	2,916	1,033	1,544	19p13.3	ubiquinol-cytochrome c reductase subunit
Hs.661	*NDUFB7*	1,845	2,416	0,884	1,272	19p13.12-p13.11	NADH dehydrogenase (ubiquinone) 1 beta subcomplex, 7
Hs.36992	-	4,848	3,213	2,277	1,684	19	
Hs.250615	*CYP2A7*	1,778	3,718	0,830	1,895	19q13.2	cytochrome P450, subfamily IIA, polypeptide 7
**Hs.356727**	***FKBP1A***	**1,918**	**4,022**	**0,940**	**2,008**	**20p13**	**FK506 binding protein 1A**
**Hs.305890**	***BCL2L1***	**6,941**	**11,508**	**2,795**	**3,525**	**20q11.21**	**BCL2-like 1, *Bcl-X***
Hs.273385	*GNAS*	1,764	2,059	0,819	1,042	20q13.2-q13.3	GNAS complex locus
Hs.284380	*GGT1*	1,784	2,762	0,835	1,466	22q11.23	gamma-glutamyltransferase 1

Relation of 30 genes presenting genomic values ≥ 1.7 [log_2_(G) ≥ 0,8] and expression values ≥ 2 [log_2_(E) ≥ 1]; it represents gains above 4-fold and overexpression above 2-fold. In bold, genes located in amplicons. **G**, Genomic values; **E**, Expression values.

### Amplicons on chromosomes 20, 2 and 8

**Figure 3:**
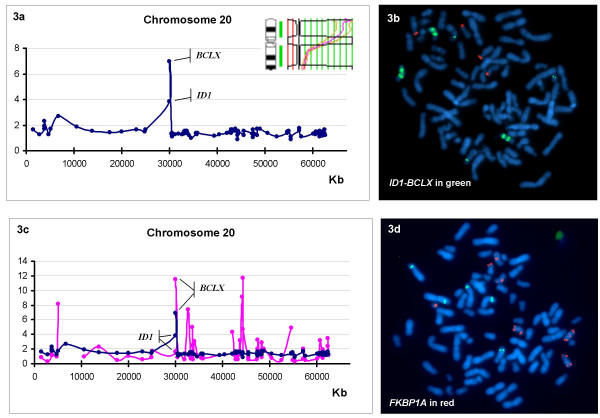
**a) **Microarray genomic values for chromsome 20. Note that the entire chromosome 20 has been gained and that, as CGH reveals, a peak of amplification is found at 20p. **b) **FISH with BAC RP11-243J16 (labeled in green) containing *ID1 and BCLX *genes to confirm genomic data; **c) **Graphical representation of microarray genomic (blue) and expression (pink) values as a function of gene position; **d) **FISH with BAC RP11-314N13 (labeled in red) containing *FKBP1A *gene on chromosome 20. Metaphase spreads from huT78 cells were prepared by standard cytogenetic methods. Gene-specific BAC clones were selected from the EnsEMBL database. Clones were labeled with SpectrumGreen-dUTP or SpectrumOrange-dUTP (Vysis) by nick translation. Dual-color hybridizations were performed at 37°C for 14–16 h and slides were washed and examined using an Olimpus AX60 epifluorescence microscope. The specificity and location of each probe was previously confirmed by FISH on normal metaphases prior to hybridization on huT78 cells.

Focusing on chromosome 20, the genomic structure of amplicon at 20p is not continuous, showing a peak of amplification around 30 Mb on the genes *ID1 *and *BCL2L1 *(*BCLX*) located at 20q11 (Table 2, Figure 3a). Both genes are close together (60 Kb apart) and are contained in the same BAC (RP11-243J16). This BAC was labeled and used as a probe for FISH on metaphase spreads of the cell line showing an extremely high amplification of this genomic fragment (figure 3b). Surprisingly, not all genes that are highly gained are also overexpressed when compared to the expression of normal T-lymphocytes. For example, although *ID1 *and *BCLX *show amplification (4 and 7 times respectively), only *BCLX *is highly expressed (Figure 3c). Other genes that are contained in the amplicon (as defined by conventional CGH) and that showed an increased copy number (some of them also overexpressed), such as *FKBP1A*, *CDC25B *and *PCNA*, were also probed by FISH using BACs RP11-314N13, RP5-1009E24 and RP4-746J20, respectively (Figure 3d). These genes are also gained but not in such a high amplification level as *BCLX*. These data are observed in their genomic values (Table 2), thus FISH with the corresponding BACs confirmed these results.

**Table 2:** Genes in amplicons of chromosome 2, 8 and 20.

**Genes in amplicon of Chromosome 20**							
	**Unigene ID**	**Gene symbol**	Position (Kb)*	**Genomic values**	**Expression values**	Genetic data**	Expression data**

*20p13*	**Hs.356727**	***FKBP1A***	-	**1,918**	**4,022**	**0,940**	**2,008**
	Hs.155140	*CSNK2A1*	451	n.d.	1,678	n.d	0,747
	Hs.156114	*PTPNS1*	1,505	1,635	0,868	0,709	-0,205
	Hs.26045	*PTPRA*	2,832	1,279	0,251	0,355	-1,997
	Hs.89648	*AVP*	3,051	n.d.	1,274	n.d	0,349
	**Hs.85004**	***CENPB***	**3,753**	**1,703**	**1,169**	**0,768**	**0,226**
	**Hs.153752**	***CDC25B***	**3,765**	**2,122**	**1,761**	**1,079**	**0,811**
	Hs.80905	*RASSF2*	4,748	1,25	0,981	0,322	-0,028
	**Hs.78996**	***PCNA***	**5,083**	**1,694**	**8,187**	**0,760**	**3,033**
	Hs.2281	*CHGB*	5,880	n.d.	0,855	n.d	-0,226
	**Hs.73853**	***BMP2***	**6,737**	**2,694**	**n.d.**	**1,430**	**n.d**
	**Hs.91143**	***JAG1***	**10,606**	**1,89**	**0,953**	**0,918**	**-0,069**
	Hs.44296	*FLJ22324*	13,761	1,543	2,292	0,626	1,197
	Hs.82306	*ADF*	17,538	1,43	0,475	0,516	-1,075
	Hs.268281	*CRN*	20,003	1,458	1,034	0,544	0,048
	Hs.2030	*THBD*	23,016	1,655	0,572	0,727	-0,806
	Hs.1872	*PCK1*	24,899	1,482	0,824	0,568	-0,279
	Hs.274264	*VSX1*	25,044	1,67	1,682	0,740	0,750
	**Hs.75424**	***ID1***	**29,981**	**3,825**	**1,640**	**1,935**	**0,714**
*20q11*	**Hs.305890**	***BCL2L1***	30,040	**6,941**	**11,508**	**2,795**	**3,525**

**Genes in amplicon of chromosome 2**							

	**Unigene ID**	**Gene symbol**	Position (Kb)*	**Genomic values**	**Expression values**	Genomic data**	Expression data**

*2q34*	Hs.54089	*BARD1*	218,722	0,734	1,646	-0,446	0,719
	**Hs.84981**	***XRCC5***	**220,094**	**1,823**	**2,815**	**0,856**	**1,341**
	**Hs.162**	***IGFBP2***	**220,592**	**2,563**	**4,544**	**1,358**	**2,184**
	**Hs.107169**	***IGFBP5***	**220,635**	**2,034**	**0,820**	**1,024**	**-0,285**
	Hs.38125	*IFI75*	224,000	1,342	1,664	0,413	0,647
	Hs.309943	*SP140*	224,057	1,45	0,646	0,536	-0,631
	Hs.83583	*ARPC2*	226,590	1,058	0,648	0,081	-0,626
	Hs.166068	*VIL1*	226,796	1,025	1,463	0,036	0,549
	**Hs.82568**	***CYP27A1***	**227,154**	**1,676**	**0,764**	**0,745**	**-0,388**
	**Hs.88049**	***PRKAG3***	**227,195**	**2,091**	**0,436**	**1,064**	**-1,196**
	**Hs.153003**	***STK16***	**227,618**	**1,657**	**1,272**	**0,729**	**0,347**
	**Hs.75318**	***TUBA1***	**227,622**	**1,631**	**5,622**	**0,706**	**2,491**
	**Hs.77768**	***DNAJB2***	**227,652**	**1,82**	**0,841**	**0,864**	**-0,250**
	**Hs.89655**	***PTPRN***	**227,662**	**1,659**	**1,447**	**0,730**	**0,533**
	Hs.48291	*PDE6D*	227,901	1,122	0,520	0,166	-0,943
	Hs.1734	*INHA*	228,352	1,187	0,849	0,247	-0,237
	Hs.198	*PAX3*	230,984	1,677	1,504	0,746	0,589
	Hs.78946	*CUL3*	233,252	1,18	0,997	0,239	-0,004
	Hs.75498	*SCYA20*	236,823	1,347	0,293	0,430	-1,769
	Hs.91400	*HDAC4*	243,313	1,26	0,543	0,333	-0,881
	Hs.36587	*PPP1R7*	244,757	0,907	1,859	-0,141	0,895
*2q37*	Hs.5345	*RNPEPL1*	245,348	1,488	0,949	0,573	-0,076
*2q35*	**Hs.82793**	***PSMB3***	-	**1,794**	**3,874**	**0,843**	**1,954**

**Genes in amplicon of chromosome 8**							

	**Unigene ID**	**Gene symbol**	Position (Kb)*	**Genomic values**	**Expression values**	Genomic data**	Expression data**

*8q23*	Hs.114218	*FZD6*	105,144	0,980	0,621	-0,030	-0,717
	Hs.86905	*ATP6C*	105,407	1,067	0,918	0,094	-0,123
	Hs.82173	*TIEG*	105,820	1,277	0,842	0,353	-0,248
	Hs.94262	*p53R2*	106,237	1,324	0,564	0,369	-0,831
	Hs.2463	*ANGPT1*	109,654	1,745	n.d	0,803	n.d.
	Hs.106673	*EIF3S6*	111,104	1.170	0.402	0.227	-1.314
	Hs.58189	*EIF3S3*	119,873	1,097	0,566	0,130	-0,885
	Hs.184161	*EXT1*	121,031	1,260	3,592	0,333	1,845
	Hs.174185	*ENPP2*	122,788	1,223	0,891	0,290	-0,167
	Hs.12940	*ZHX1*	127,568	1,212	1,309	0,277	0,389
	Hs.61661	-	127,822	1,101	1,318	0,139	0,399
	Hs.181107	*ANXA13*	127,989	1,333	0,655	0,415	-0,611
	Hs.344478	*KIAA0196*	129,211	1,302	n.d	0,381	n.d
	**Hs.79070**	***MYC***	**132,293**	**1,883**	**1,597**	**0,853**	**0,630**
	**Hs.305916**	***TG***	**136,747**	**1,441**	**1,711**	**0,527**	**0,775**
	**Hs.75789**	***NDRG1***	**137,117**	**1,617**	**1,140**	**0,693**	**0,190**
	**Hs.157240**	***MGC4737***	**143,611**	**1,722**	**0,828**	**0,784**	**-0,273**
	**Hs.740**	***PTK3***	**144,545**	**1,896**	**0,939**	**0,914**	**-0,487**
	**Hs.77667**	***LY6E***	**146,427**	**1,595**	**2,410**	**0,674**	**1,269**
	**Hs.301118**	***CYP11B1***	**146,498**	**1,619**	**1,227**	**0,695**	**0,295**
	Hs.348605	-	146,918	1,309	0,826	0,388	-0,276
	Hs.264428	*TSTA3*	147,413	1,365	0,693	0,449	-0,530
	Hs.223241	*EEF1D*	147,485	1,554	3,142	0,636	1,651
	Hs.323834	*NFKBIL2*	147,600	1,375	4,436	0,459	2,149
	Hs.339697	*LOC51160*	147,616	1,418	1,546	0,504	0,629
	Hs.31442	*RECQL4*	147,653	1,458	3,419	0,544	1,774
	Hs.12271	-	147,813	1,640	2,034	0,714	1,024
	Hs.92679	-	147,876	1,178	1,510	0,236	0,595
*8q24*	**Hs.331601**	***-***	**147,927**	**2,138**	**1,077**	**1,096**	**0,107**

Areas of genomic gain presenting the highest values are shown in bold. Note that in 20p amplicon, the entire region is gained, but only the highest values are indicated. Underlined are gained and overexpressed genes. *Map location according to the EnsMBL database (with exception of *FKBP1A *and *PSMB3 *genes that are FISH mapped); **Log_2 _values; **n.d. **No data.

Using CGH, amplicon in chromosome 2 is narrower than amplicon in chromosome 20 (Figure 1). In fact it seems to be restricted to a few genes if genomic data are examined (Table 2). Only two areas, one around 220 and the other around 227 Mb, present the highest genomic values (shadowed in table 2), with 4 genes overexpressed: *XRCC5 *(2q35) and *IGFBP2 *(2q33-24) around 220 Mb, *TUBA1 *(tubulin, alpha 1) around 227 Mb and *PSMD3 *mapped by FISH at 2q35. Amongst these, *IGFBP2 *is the most highly overexpressed (Table 2).

Amplicon in chromosome 8 is located at 8q23-q24, where the gene *cMYC *is located. This gene was found as amplified in other tumors and in this study we show that it is also amplified in the cell line huT78. *cMYC *is not however highly expressed in this cell line (Table 2). Chromosome 8 amplicon affects a wide region, from 132 to 146 Mb (shadowed in table 2), but only one gene located within this area is overexpressed, *LY6E *(*lymphocyte antigen 6 complex*, *locus E*). This gene is upregulated by all-trans-retinoic acid (ATRA), a differentiation inducer capable of causing clinical remission in about 90% of patients with acute promyelocytic leukemia. Other genes located in the amplicon that have not been analyzed in this study could also be important.

### Analyses of the amplicon candidate genes in primary T-cell lymphomas and cell lines

Expression of the relevant genes found in the amplicons that were gained in this T-cell line were examined in a series of 39 primary T-cell lymphomas and 3 T-cell lines (Jurkat, Molt 16 and Karpas 45) [19]. Three genes located in 20q amplicon were overexpressed in the majority of primary tumors and stablish cell lines. *FKBP1A *and *PCNA *were overexpressed in 89,7 and 71,8% respectively (35 and 28 out of 39) of the primary tumors and in all the three cell lines analyzed. In addition, *BCLX *was overexpressed in 64,1% (25/39) of the primary tumors and in Jurkat cell line. Regarding genes in amplicons of chromosome 2 and 8, *IGFBP2 *was only overexpressed in 15,4% (6/39) of the tumors and *cMYC *is not highly expressed in any of them. However, both genes were overexpressed in the three cell lines.

## Discussion

Conventional chromosome CGH of the T-cell line huT78 showed genomic patterns of copy number changes affecting most of the chromosomes. Three highly amplified regions were detected using this technique. Nevertheless, resolution of chromosome CGH is no less than 2 Mb for copy number gains and is a function of both amplicon size and copy number gains [20].

In order to precisely define regions of high-level amplification and to identify amplified and highly expressed genes contained in the amplicons of the T-cell line huT78, we performed cDNA microarray CGH and expression profiling of the cell line. For this purpose, we used the CNIO Oncochip containing 7.657 different sequence-validated cDNA clones that represent known genes and expressed sequence tags (ESTs) related to the tumoral process, and tissue specific genes.

Recently, several studies have shown the utility of cDNA microarray CGH for studing gene copy changes in various types of tumors, and the usefulness of defining amplicon boundaries at high resolution (gene level) to assist in locating and identifying candidate oncogenes [4-11]. However, to date, no such studies on T-cell lymphomas have been performed. In the present study, we determined the precise locations of gene amplifications in the T-cell line huT78 by the use of array-based CGH on cDNA microarrays. We observed that, although chromosome CGH detected amplicons of different size (as for example the amplicons on chromosomes 20 and 2), the structure of those amplicons is more complex when it is analyzed at high resolution. Amplicon in chromosome 20 extends along all p arm when detected by use of chromosome CGH. Instead, several peaks of amplification, different in magnitude, appear at 3, 5–6, 10 and 29–30 Mbs when analyzed at gene-by-gene level. Amplicon in chromosome 2, although showing a narrower shape by chromosome CGH than that of chromosome 20, also presented a complex structure at high resolution, with two peaks of amplification around 220 and 227 Mbs.

By analyzing mRNA levels in parallel, we observed that not all genes that are amplified are also overexpressed. Thus, by analyzing the expression of the genes contained in the amplicons, we selected several genes whose expression seemed to respond to gene copy number gains. We showed that only a few of the genes contained in the amplicon are highly gained and also overexpressed. In this way we could select the candidate genes to study further that could be involved in the tumorogenesis of the T-cell lymphomas.

As stated before [7], however, the elevated expression of an amplified gene cannot alone be considered strong independent evidence of a candidate oncogene's role in tumorigenesis. Thus, we examined the expression of the selected candidate genes in a series of 39 primary T-cell lymphomas and 3 T-cell lines (Jurkat, Molt 16 and Karpas 45) [19]. Three genes located in 20q amplicon were overexpressed in the majority of primary tumors and stablish cell lines. *FKBP1A*, *PCNA *and *BCLX *were overexpressed in 90–65% of the primary tumors. *FKBP1A *is a receptor for rapamicin as well as for FK506, a powerful immunosuppressant in T-cells, and *PCNA *is involved in the control of eukaryotic DNA replication and may have a role in DNA repair synthesis. *BCLX *is involved in both positive and negative regulation of programmed cell death. Thus, these three genes, could be significant in the development of T-cell lymphomas.

When the candidate genes located in amplicons of chromosome 2 and 8 were examined, we observed that *IGFBP2 *was only overexpressed in a low proportion of the primary tumors (15%) and *cMYC *is not highly expressed in any of them. Curiously, both genes were overexpressed in the three cell lines suggesting that they could be of relevance in the maintenance of the cell lines.

## Conclusions

We have identified 3 amplicons by CGH in the T-cell line huT78. By cDNA microarray CGH. We have narrowed down the regions and selected some amplified and overexpressed genes: *BCLX*, *PCNA *and *FKBP1A*. These genes are also overexpressed in 65–90% of a set of 39 T-cell lymphomas and 3 cell lines, while *IGFBP2 *and *cMYC *are only overexpressed in T-cell lines. Our data suggest a different role of these genes in such processes. New studies with more clones covering these amplicons can help us to better identify relevant genes in T-cell lymphomas.

## Authors' contributions

BM carried out the genomic and RNA hybridizations of the cell line onto the microarrays, performed FISH experiments, analyzed the results and drafted the manuscript. BM-D participated in the design of the study and in the analysis and discussion of the results. MC performed the amplification of the RNA and hybridization onto the microarray to carry out expression studies of primary tumors. VF performed nucleic acid extractions for hybridizations onto the microarray, grew and label the BAC clones for FISH assays and performed CGH. RD-U carried out statistical analysis of the microarray results. JB conceived of the study, and participated in its design and coordination. All authors read and approved the final manuscript.

## Supplementary Material

Additional File 1 This file contains row genomic and expression data for each gene. Each gene is identified by a unique clone identification (Clone ID), the corresponding gene symbol (Gene Symbol) and the Unigene number (Unigene ID). Localization of all clones are also shown (map positions of the cDNA clones were obtained from the EnsEMBL database, as referred in the text). Click here for file
